# A Study of Faster-Z Evolution in the Great Tit (*Parus major*)

**DOI:** 10.1093/gbe/evaa044

**Published:** 2020-03-02

**Authors:** Kai Hayes, Henry J Barton, Kai Zeng

**Affiliations:** e1 Department of Animal and Plant Sciences, University of Sheffield, United Kingdom; e2 Organismal and Evolutionary Biology Research Program, University of Helsinki, Finland

**Keywords:** effective population size, positive selection, genetic drift, Z chromosome, sex chromosomes

## Abstract

Sex chromosomes contribute substantially to key evolutionary processes such as speciation and adaptation. Several theories suggest that evolution could occur more rapidly on sex chromosomes, but currently our understanding of whether and how this occurs is limited. Here, we present an analysis of the great tit (*Parus major*) genome, aiming to detect signals of faster-Z evolution. We find mixed evidence of faster divergence on the Z chromosome than autosomes, with significantly higher divergence being found in ancestral repeats, but not at 4- or 0-fold degenerate sites. Interestingly, some 4-fold sites appear to be selectively constrained, which may mislead analyses that use these sites as the neutral reference (e.g., *d*_N_*/d*_S_). Consistent with other studies in birds, the mutation rate is significantly higher in males than females, and the long-term Z-to-autosome effective population size ratio is only 0.5, significantly lower than the expected value of 0.75. These are indicative of male-driven evolution and high variance in male reproductive success, respectively. We find no evidence for an increased efficacy of positive selection on the Z chromosome. In contrast, the Z chromosome in great tits appears to be affected by increased genetic drift, which has led to detectable signals of weakened intensity of purifying selection. These results provide further evidence that the Z chromosome often has a low effective population size, and that this has important consequences for its evolution. They also highlight the importance of considering multiple factors that can affect the rate of evolution and effective population sizes of sex chromosomes.

## Introduction

Sex chromosomes play a significant role in key evolutionary processes such as speciation and adaptation (Charlesworth et al. 1987; Vicoso and Charlesworth 2006). Understanding this phenomenon is essential for developing our understanding of fundamental aspects of evolution. Several theories suggest that evolution could occur more rapidly on the sex chromosomes than the autosomes (Haldane 1924, 1926; Charlesworth et al. 1987)*.* This is commonly known as the faster-Z effect (or faster-X for male heterogametic species such as humans) and is traditionally attributed to the possibility that positive selection could be more effective on the Z chromosome (Haldane 1924, 1926; Charlesworth et al. 1987). However, if the rate of evolution is taken to be the speed at which allele frequencies change over time, then it is also possible for other factors such as increased genetic drift or the decreased efficacy of purifying selection to contribute to a faster rate of evolution on the Z chromosome.

Theoretically, there are several reasons to expect either increased efficacy of positive selection or increased genetic drift on the Z chromosome. Firstly, there is only a single copy of the Z chromosome in the heterogametic sex, whereas autosomes are always present in pairs. On the one hand, this allows for greater expression of recessive mutations on the Z chromosome, which could increase the efficacy of selection on recessive beneficial variants, leading to faster rates of adaptation (Haldane 1924, 1926; Charlesworth et al. 1987). On the other hand, it reduces the effective population size (*N*_e_) of the Z chromosome (*N*_eZ_) to ¾ of that of the autosomes (*N*_eA_), which could increase the amount of genetic drift (Charlesworth 2009; Ellegren 2009). This could result in relaxed purifying selection on deleterious mutations and accelerated rates of fixation of mildly deleterious mutations on the Z chromosome (Vicoso and Charlesworth 2006; Charlesworth 2009).

Sex chromosomes differ from the autosomes in their response to demographic events. Theoretical studies suggest that due to its lower effective population size, the Z chromosome converges to the new equilibrium at a higher rate than autosomes after a population size change, causing transient changes in *N*_eZ_*/N*_eA_ (Pool and Nielsen 2007)*.* Furthermore, male- or female-biased migration can also alter the sex ratio of a population, and consequently change the value of *N*_eZ_*/N*_eA_ (Laporte and Charlesworth 2002). Failing to control for the effects of demography may lead to biased estimates of *N*_eZ_*/N*_eA_ (Zeng et al. 2019).

Similarly, the type of mating system can influence effective population size ratios. Polygyny is common in the natural world, and results in increased variance in male reproductive success compared with female (Ellegren 2009). This has opposite effects on the X and Z chromosomes, increasing the *N*_eX_*/N*_eA_ ratio and decreasing the *N*_eZ_*/N*_eA_ ratio (Vicoso and Charlesworth 2009; Webster and Wilson Sayres 2016).

Additionally, the mutation rate can vary between the sex chromosomes and the autosomes. Spermatogenesis usually requires more cell divisions than oogenesis, which increases the mutation rate in the male germline (Drake et al. 1998; Vicoso and Charlesworth 2006). Again this has opposing effects on the X and Z chromosomes, decreasing the mutation rate on the X relative to the autosomes, and increasing the mutation rate on the Z relative to the autosomes. These phenomena are known as male-driven evolution (Li et al. 2002; Ellegren 2007).

In reality, some, or all, of these factors may act simultaneously, and evolutionary patterns are determined by the relative importance of the contributing factors. It can therefore be challenging to tease apart the potential causes of faster-X or faster-Z evolution, but recent advances in sequencing technologies and the increased availability of such data have opened up new opportunities to empirically test these ideas. However, studies to date paint a complicated picture.

Much of the empirical work thus far has focused on the X chromosome, finding mixed results. In *Drosophila*, several studies comparing the ratio of nonsynonymous to synonymous substitutions (*d*_N_*/d*_S_) have found evidence of faster divergence on the X chromosome (Counterman et al. 2004; Musters et al. 2006), whereas others have not (Betancourt et al. 2002; Thornton et al. 2006; Vicoso et al. 2008). The problem with this approach is that it is difficult to determine whether the increase in the *d*_N_*/d*_S_ ratio is due to increased efficacy of positive selection or relaxation of purifying selection (Meisel and Connallon 2013; Kousathanas et al. 2014). A better approach is to analyze both polymorphism and divergence data simultaneously using the McDonald–Kreitman approach (McDonald and Kreitman 1991). This allows the estimation of the adaptive substitution rate (e.g., as measured by α or ω_a_) while controlling for the impact of purifying selection (Eyre-Walker and Keightley 2009; Tataru et al. 2017; Barton and Zeng 2018). However, results here have also been mixed, with some studies finding faster adaptive evolution on the X (Baines et al. 2008; Mackay et al. 2012; Charlesworth et al. 2018) and others not (Connallon 2007). The strongest evidence of faster adaptive evolution on the X chromosome in *Drosophila* is found for genes that are more strongly expressed in males, which is consistent with theoretical predictions (Meisel and Connallon 2013; Charlesworth et al. 2018). In vertebrates, there is evidence of a faster rate of adaptive evolution on the X chromosome in chimpanzees (Hvilsom et al. 2012), mice (Kousathanas et al. 2014), and some rabbits (Carneiro et al. 2012).

Relatively less work has looked at the possibility of faster evolution on the Z chromosome, although theoretically there should be substantial similarity between faster-X and faster-Z effects. Existing work on the Z chromosome suggests there may be some important differences. Most previous studies focus on comparing the rate of divergence (e.g., as measured by *d*_N_*/d*_S_), and the Z chromosome has been found to evolve faster in birds, *Lepidoptera*, and some snakes (Borge et al. 2005; Mank, Nam, et al. 2010; Vicoso et al. 2013; Sackton et al. 2014; Wang et al. 2014; Wright et al. 2015; Xu, Auer, et al. 2019; Xu, Wa Sin, et al. 2019). Fewer studies have compared the rate of adaptive substitution between the Z chromosome and the autosomes, and of these a faster rate of adaptive evolution on the Z chromosome has been observed in silkmoths (Sackton et al. 2014) and *Heliconius* butterflies (Pinharanda et al. 2019), but not in satyrine butterflies (Rousselle et al. 2016).

Interestingly, *N*_eX_*/N*_eA_ ratios are frequently larger than the expected null value of 0.75, whereas *N*_eZ_*/N*_eA_ ratios are frequently lower (Charlesworth 2009; Ellegren 2009; Mank, Vicoso, et al. 2010). This points to an important difference in evolutionary dynamics between the X and Z chromosomes, most likely caused by the prevalence of polygyny in nature, which leads to high variance in male reproductive success (Ellegren 2009; Corl and Ellegren 2012; Oyler-McCance et al. 2015; Wright et al. 2015). This has important evolutionary consequences. Several studies in birds have shown that a faster rate of divergence on the Z chromosome is probably due to increased genetic drift because of the especially low *N*_eZ_*/N*_eA_ ratio (Mank, Nam, et al. 2010; Wang et al. 2014; Wright et al. 2015; Xu, Wa Sin, et al. 2019). In contrast, on the X chromosome drift may be comparatively less important, as the *N*_eX_*/N*_eA_ ratio often approaches 1, which allows other factors such as the increased expression of recessive mutations to become more prominent (Meisel and Connallon 2013; Kousathanas et al. 2014; Charlesworth et al. 2018).

However, there remains a need to study faster-X and -Z effects in different species or groups to better understand the generality of previous findings and how different factors interact to produce these effects. The great tit (*Parus major*) is closely related to several other species in which the faster-Z effect has been studied such as the zebra finch and collared flycatcher (Mank et al. 2007; Mank, Nam, et al. 2010; Mank, Vicoso, et al. 2010), but is known to differ from these in key parameters that are important in faster-Z evolution. For instance, compared with the zebra finch, the great tit’s effective population size is about two to three times smaller, and its population size is more stable in the recent past (Barton HJ and Zeng K, in preparation; Corcoran et al. 2017). Great tits are also one of the less promiscuous passerine species (Dhondt 1987; Gohli et al. 2013), which may imply that *N*_eZ_*/N*_eA_ is less affected by polygyny. Thus, the great tit presents an interesting system in which to study the faster-Z effect.

In the present study, the evidence for a faster rate of evolution on the Z chromosome in the great tit is assessed, and its potential causes investigated, by combining results from several analyses. In particular, recently published models by Barton and Zeng (2018) and Zeng et al. (2019) are used, as they can provide estimates of several parameters known to be important in faster-Z evolution, including *N*_eZ_/*N*_eA_, past demography, difference in the mutation rate between the Z and autosomes, the distribution of fitness effects of new mutations, and efficacy of selection.

## Materials and Methods

### Data

Both intra- and inter-specific genomic data were used in this study. Full details of sequencing, annotation, and filtering are described in Corcoran et al. (2017) and Barton and Zeng (2019), but key points are summarized here. The polymorphism data set consisted of ten European great tit males, from different populations, sequenced to high coverage (44×) as described in Corcoran et al. (2017). We obtained the VCF file of filtered single-nucleotide polymorphisms (SNPs) for this data set as used in Barton and Zeng (2019). Briefly, this VCF was generated using the GATK (version 3.4) workflow (McKenna et al. 2010; DePristo et al. 2011; Van der Auwera et al. 2013), and SNPs passing the 99% tranche cutoff following variant quality score recalibration were retained. Additionally, SNPs with coverage more than twice, or less than half, the mean coverage of 44×, variants in repeat regions identified by RepeatMasker (http://www.repeatmasker.org/; last accessed August 29, 2019), multiallelic sites, and sites where the total number of alleles was <20 were excluded. Note that the level of differentiation between European great tit populations is very low (Kvist et al. 1999; Laine et al. 2016) and the “scattered sampling” strategy employed in Corcoran et al. (2017) should additionally help to remove any residual effects of population structure (Wakeley 1999).

We identified 0-fold degenerate sites (henceforth 0-fold sites) and 4-fold degenerate sites (henceforth 4-fold sites) using the great tit coding sequence fasta file (version 1.03) (available from: ftp://ftp.ncbi.nlm.nih.gov/genomes/all/GCF/001/522/545/GCF_001522545.1_Parus_major1.0.3/GCF_001522545.1_Parus_major1.0.3_cds_from_genomic.fna.gz; last accessed August 29, 2019), and ancestral repeat regions using the coordinates of conserved LINE elements identified in Barton and Zeng (2019). Mutations at 0-fold sites alter amino acid sequences and thus are more likely to be under selection, whereas mutations at 4-fold sites do not alter amino acid sequences so are putatively neutral. Ancestral repeats have no known function, and are often assumed to be neutral. Thus, the data represent both selected and putatively neutral types of site. Information on the numbers of sites analyzed can be found in supplementary table S1, Supplementary Material online.

### Divergence

We obtained a three-way multispecies whole-genome alignment from Barton and Zeng (2019). The alignment consisted of the reference genomes of great tit (*P.* *major*) (version: 1.04), collared flycatcher (*Ficedula albicollis*) (version: FicAlb1.5), and zebra finch (*Taeniopygia guttata*) (version: TaeGut3.2.4). The alignment was generated using LastZ (Harris 2007) to create pairwise genome alignments for the great tit and collared flycatcher against the zebra finch genome. The pairwise alignments were then chained and netted using axtChain and chainNet, respectively (Kent et al. 2003). The resulting pairwise alignments were then filtered to ensure single coverage of the reference genome using “single_cov2.v11” from the MULTIZ package and aligned using MULTIZ (Blanchette et al. 2004). Only regions where all three species were successfully aligned were used in the analyses.

From this alignment, we generated FASTA files of concatenated sites from each site class of interest (0-fold sites, 4-fold sites, and sites in ancestral repeats) and used APE (Paradis et al. 2004) in R (https://www.r-project.org/; last accessed August 29, 2019) to generate a pairwise distance matrix with the function “dist.dna” with “model=K80.” The pairwise distance matrix was used to obtain branch-specific divergence estimates for the great tit lineage. Divergence was calculated for 0-fold sites, 4-fold sites, and ancestral repeat regions on both the Z chromosome and the autosomes. The divergence estimates for 0- and 4-fold sites were used to calculate the nonsynonymous to synonymous substitution ratio (*d*_0_*/d*_4_). The 0-fold to ancestral repeat divergence ratio (*d*_0_*/d*_AR_) was also calculated.

### Polymorphism-Based Statistics

Single-nucleotide polymorphisms within the sample of ten great tits were used to calculate nucleotide diversity π (Tajima 1983), Watterson’s θ (Watterson 1975), and Tajima’s *D* (Tajima 1989) at 0-fold sites, 4-fold sites, and ancestral repeat regions on both the Z chromosome and the autosomes. All calculations were performed using Python 3 and the packages PyVCF (available from: https://github.com/jamescasbon/PyVCF, last accessed August 30, 2019) and SeqIO in Biopython (Cock et al. 2009). To obtain per site estimates of nucleotide diversity and Watterson’s θ, we divided our estimates by the number of sites for each site class that were successfully called and passed filtering in the genotype calling conducted in Barton and Zeng (2019). These numbers of “callable sites” were also used to obtain per site estimates in the *VarNe* (Zeng et al. 2019) and *anavar* (Barton and Zeng 2018) analyses described below.

### Estimating *N*_eZ_*/N*_eA_, Past Demography, and the Mutation Rate

As mentioned in the Introduction, failing to control for recent demographic changes can lead to biased estimates of *N*_eZ_/*N*_eA_. A recent study has shown that this can be alleviated by fitting an explicit demographic model to polymorphism data collected from the Z chromosome and autosomes simultaneously (Zeng et al. 2019). In addition, this new approach, implemented in the software *VarNe*, can also produce an estimate of *u*_Z_/*u*_A_, where *u*_Z_ and *u*_A_ are the mutation rate per site per generation on the Z chromosome and autosomes, respectively. Hence, it provides an alternative way of detecting evidence of male-driven evolution that is semi-independent from the classical, divergence-based approach (Li et al. 2002; Ellegren 2007).

We only used polymorphic sites in putatively neutral ancestral repeat regions on the Z chromosome and autosomes for this analysis, to avoid the confounding effects of selection. *VarNe* is capable of accepting multiple site frequency spectra (SFS) for each locus (here the Z chromosome was regarded as a locus, and the autosomes were regarded as the other locus). For each locus, we entered two SFSs, one unfolded SFS containing sites for which the ancestral state could be inferred from the multispecies alignment using maximum parsimony (where all outgroups were required to match either the reference, or the alternate, allele in the great tit in order to assign it as ancestral), and one folded SFS containing the rest of the sites. This procedure maximizes the amount of data the program could use, therefore increasing the accuracy of the estimates. Inferring ancestral states using parsimony is known to be error prone, which can distort the site frequency spectrum leading to the inaccurate estimation of population genetic parameters (Hernandez et al. 2007; Barton and Zeng 2018). *VarNe* deals with this problem by introducing polarization error as free parameters to be estimated from data (ε_Z_ and ε_A_ for the Z-linked and autosomal data, respectively). This approach has been used in multiple previous studies (Gutenkunst et al. 2009; Glémin et al. 2015; Barton and Zeng 2018).

We considered a demographic model with a one-step change in population size; increasing the number of epochs to three did not significantly improve the fit. Specifically, the model assumes that the effective population size on the Z chromosome before the recent population size change is *N*_eZ_, and that this epoch extends infinitely into the past (see supplementary fig. S1, Supplementary Material online, for a graphical representation of the model and its parameters). Using *N*_eZ_ as the “reference” effective population size, we define θ_Z_ = 4 *N*_eZ_*u*_Z_, θ_A_ = 4 *N*_eZ_*u*_A_, τ = *T*/(2*N*_eZ_), where *T* is the number of generations before the present when the population size change took place. Because both θ_Z_ and θ_A_ are defined in terms of *N*_eZ_, they are directly comparable and their ratio provides an estimate of *u*_Z_/*u*_A_. The ratio of effective population size in the ancestral epoch (i.e., before the population size change) is *N*_eZ_/*N*_eA_ = 1/*f*, where *f* is a free parameter to be estimated from the data. To allow for changes in the ratio of effective population size induced by sex-biased demographic factors (Laporte and Charlesworth 2002), the model assumes that, after the population size change, the effective size on the Z chromosome becomes *g*_Z_*N*_eZ_ and that on the autosomes becomes *g*_A_*fN*_eZ_, such that the new ratio of effective population size is *g*_Z_/(*g*_A_*f*).

Two reduced models were fitted to the data by adding constraints to the full model—in the first model, we required the mutation rate to be the same on the Z chromosome and autosomes; in the second case, *N*_eZ_*/N*_eA_ was fixed at 0.75. Likelihood ratio tests could then be conducted comparing these reduced models to the full model to investigate: 1) whether the mutation rate was significantly different between the Z chromosome and the autosomes and 2) whether the *N*_eZ_*/N*_eA_ ratio was significantly different from 0.75. These results were further corroborated by bootstrapping analyses (see below).

### Estimating the Efficacy of Selection

We compared the efficacy of both positive and negative selection between the Z chromosome and autosomes by using a McDonald–Kreitman approach. We began by using the “neutralSNP_vs_selectedSNP” model implemented in the program *anavar* (Barton and Zeng 2018) to analyze polymorphism data. This model can produce maximum likelihood estimates (MLEs) of the distribution of fitness effects (DFE) for 0-fold variants while controlling for the confounding effects of demography. Although this model requires the use the unfolded SFS, its built-in polarization error correction method performs well (Barton and Zeng 2018), even in the presence of positively selected variants (supplementary table S2, Supplementary Material online). We inferred the DFE for 0-fold variants on the Z chromosome and the autosomes, separately, using either 4-fold sites or ancestral repeats on the same chromosome type as the neutral reference.

We assumed a discrete DFE model in which the fitness effect of a new 0-fold mutation could fall into one of *c* site classes. Each site class has its own scaled selection coefficient γ (4 *N*_e_*s*, where *s* is the selection coefficient, and the fitnesses of the wild-type, heterozygote, and mutant homozygote genotypes are 1, 1 + *s*, and 1 + 2 *s*, respectively). The scaled mutation rate θ (4 *N*_e_*u*, where *u* is the mutation rate per site per generation) is the same between 0-fold sites and sites in the neutral region. A DFE with two selected site classes (*c *= 2) was the best fit for the data and increasing the number of site classes to three did not significantly improve the fit. This DFE was then used to calculate the proportion of substitutions fixed by positive selection (α) (e.g., equations 18 and 19 in Barton and Zeng 2018) and the (relative) rate of adaptive substitution relative to the neutral rate of substitution (ω_a_) (Gossmann et al. 2012). We also repeated the above analysis by assuming that the DFE follows a gamma distribution, and obtained qualitatively similar results (see below).

### Bootstrapping

95% Confidence intervals (CIs) for each analysis were obtained by analyzing 100 bootstrap replicate data sets produced by randomly resampling loci (gene or ancestral repeat, respectively) with replacement.

### Data availability

The aforementioned multispecies alignment files, VCF files, and BED files containing the coordinates of various genomic elements can be downloaded from http://zeng-lab.group.shef.ac.uk (last accessed January 10, 2020). The scripts used in the analysis can be found on https://github.com/henryjuho/hayes_et_al (last accessed January 10, 2020).

## Results

### Divergence

**Fig. 1.**
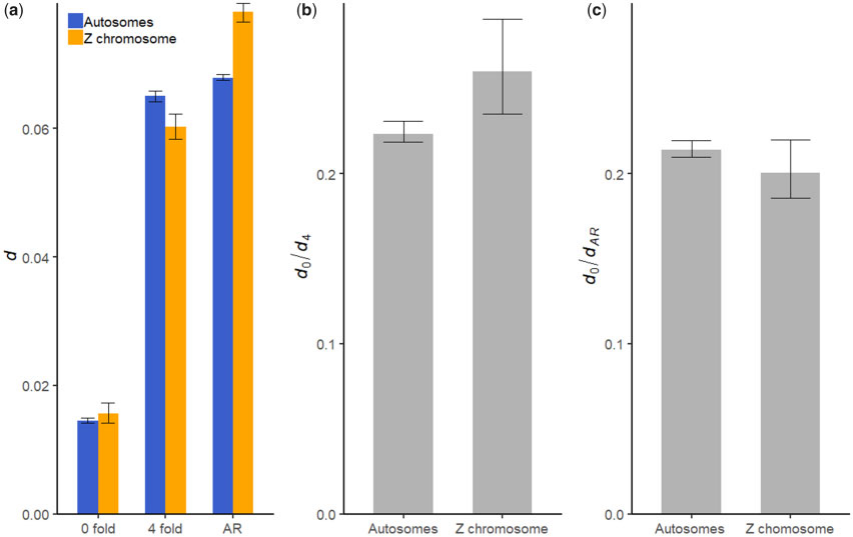
—Comparing divergence levels between the Z chromosome (blue) and the autosomes (orange) for (*a*) different regions of interest, (*b*) 0-fold versus 4-fold changes (*d*_0_*/d*_4_), and (*c*) 0-fold changes compared with changes in ancestral repeats (*d*_0_*/d*_AR_). Error bars show 95% CIs.

On both types of chromosomes, the level of divergence was significantly lower at 0-fold sites than the putatively neutral sites (4-fold and ancestral repeats; bootstrapping *P *<* *0.05; fig. 1*a*), indicating that 0-fold sites have been subject to evolutionary constraints and purifying selection. Interestingly, divergence at 4-fold sites is significantly lower than that at ancestral repeats (bootstrapping *P *<* *0.05; fig. 1*a*). A similar observation was made previously by Künstner et al. (2011), and may be indicative of selective constraints on some of the 4-fold sites.

We found no significant difference in divergence between the Z chromosome and the autosomes at 0-fold sites (bootstrapping *P *>* *0.05; fig. 1*a*). Divergence was significantly lower on the Z chromosome at 4-fold sites (bootstrapping *P *<* *0.05; fig. 1*a*), but significantly higher on the Z chromosome in ancestral repeat regions (bootstrapping *P *<* *0.05; fig. 1*a*). The reason for this inconsistency is unclear, but that some 4-fold sites appear to behave nonneutrally likely contributes and makes the observation harder to interpret. The use of 4-fold sites is further complicated by a notable difference in GC content between the Z chromosome and autosomes (44% vs. 53%), because GC content is known to be positively correlated with substitution rates in birds (Axelsson et al. 2005; Webster et al. 2006; Gossmann et al. 2014). In contrast, ancestral repeat regions have similar GC content (48.6% vs. 49.3%) on the two types of chromosomes.

Finally, the ratio of divergence rate between 0- and 4-fold sites (*d*_0_*/d*_4_) was significantly larger on the Z chromosome than the autosomes (bootstrapping *P *<* *0.05; fig. 1*b*). However, when *d*_0_ was normalized by *d*_AR_ as a control for possible differences in the mutation rate, the *d*_0_*/d*_AR_ ratio was not significantly different (bootstrapping *P *>* *0.05; fig. 1*c*). These ratios are difficult to interpret because *d*_0_ depends on the relative frequencies of neutral, beneficial, and weakly deleterious mutations (i.e., the DFE). We use the McDonald–Kreitman approach to estimate the relative contribution of both positive and negative selection in a later section.

### Polymorphism Patterns

**Fig. 2.**
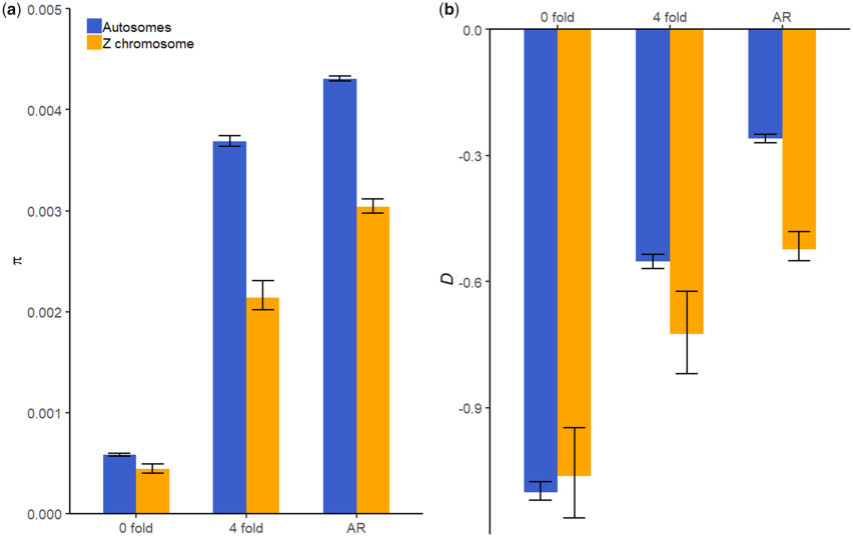
—Nucleotide diversity (*a*) and Tajima’s *D* (*b*) for different regions of the genome on both the Z chromosome (blue) and the autosomes (orange). Error bars show 95% CIs.

The level of genetic diversity was significantly lower at 0-fold sites than 4-fold sites and ancestral repeats (bootstrapping *P *<* *0.05; fig. 2*a*). This implies that 0-fold sites are under evolutionary constraints and purifying selection. This conclusion is further supported by significantly more negative Tajima’s *D* values at these sites (bootstrapping *P *<* *0.05; fig. 2*b*), and is consistent with reduced levels of divergence at these sites. The diversity level at 4-fold sites was clearly lower than that at ancestral repeats (bootstrapping *P *<* *0.05; fig. 2*a*). This may be due to linked selection having a stronger effect in reducing diversity at 4-fold sites, relative to ancestral repeats, because 4-fold sites are more tightly linked to potentially selected variants (e.g., 0-fold mutations). Alternatively, it suggests that purifying selection may have played a role in the evolution of 4-fold sites. Because *d*_4_ < *d*_AR_ (fig. 1*a*), and because linked selection does not affect the rate of substitution (Birky and Walsh 1988), the observed polymorphism and divergence patterns can be readily explained by selective constraints on some of the 4-fold sites, although this does not preclude the possibility that linked selection may have also affected the polymorphism pattern.

The genetic diversity was also significantly lower on the Z chromosome than the autosomes for all types of site (bootstrapping *P *<* *0.05; fig. 2*a*). At neutral sites, the Z chromosome to autosome diversity ratio (π_Z_/π_A_) is proportional to the *N*_eZ_*/N*_eA_ ratio, assuming that the mutation rate is the same. The π_Z_/π_A_ ratio was 0.58 (95% CI: [0.54, 0.63]) at 4-fold sites and 0.71 (95% CI: [0.68, 0.74]) in ancestral repeat regions, in both cases significantly lower than the expected null value of 0.75. Assuming that the autosomal mutation rate in the great tit is 4.6 × 10^−9^ per site per generation, the same as the collared flycatcher (Smeds et al. 2016), the autosomal effective population size estimated using π_4_ or π_AR_ is 2.01 × 10^5^ or 2.3 × 10^5^, respectively. The selected to neutral diversity ratio (π_0_/π_4_ or π_0_/π_AR_, respectively) can be used as a rough indicator of the efficacy of purifying selection. The π_0_/π_4_ ratio was 0.21 (95% CI: [0.19, 0.23]) on the Z chromosome, significantly higher than the value of 0.16 (95% CI: [0.15, 0.16]) on the autosomes. The π_0_/π_AR_ ratio was 0.14 (95% CI: [0.13, 0.17]) on the Z chromosome and was not significantly different from the value of 0.13 (95% CI: [0.13, 0.15]) on the autosomes. Again, the fact that some 4-fold sites may be under purifying selection confounds the interpretation of π_0_/π_4_, and π_0_/π_AR_ may depend on demography and the DFE in a complex way. Thus, we use a model-based approach to further test for any difference in the efficacy of selection between the two types of chromosomes below.

Negative Tajima’s *D* values at the putatively neutral ancestral repeats provide evidence of a population expansion (fig. 2*b*). This appears to have had a more significant effect on the Z chromosome, as Tajima’s *D* is significantly lower on the Z chromosome than the autosomes at these sites (bootstrapping *P *<* *0.05; fig. 2*b*). In contrast, at selected sites, there was no significant difference in Tajima’s *D* between the Z chromosome and the autosomes (bootstrapping *P *>* *0.05; fig. 2*b*). A possible explanation is that purifying selection on most of the segregating 0-fold variants is sufficiently strong that population size change has a relatively weak effect on their frequency in the population. For instance, in the deterministic limit, the frequency of deleterious mutations with additive effects on fitness is proportional to *u*/*s*, independent of the population size, where *u* is the mutation rate and *s* is the selection coefficient. This explanation is corroborated by our model-based inference of the DFE presented below.

It is known that avian chromosomes vary significantly in size, and that macrochromosomes and microchromosomes different in, for example, recombination rate, gene density, and GC content (Ellegren 2010). We recalculated all the statistics described earlier using data from autosomal macrochromosomes (chromosomes 1–12; Gossmann et al. 2014). The values of the statistics (supplementary table S3, Supplementary Material online) are very similar to those calculated on all autosomes. This is because most autosomal data are from the macrochromosomes. Considering that the models we use below are parameter-rich, we analyze data from all autosomes to enhance statistical power.

### Estimating *N*_eZ_*/N*_eA_, Past Demography, and the Mutation Rate

Maximum likelihood parameter estimates obtained by applying *VarNe* (Zeng et al. 2019) to polymorphism data on ancestral repeats are shown in table 1. The *N*_eZ_*/N*_eA_ ratio in epoch 2 (the most distant epoch) is given by 1*/f*, and the *N*_eZ_*/N*_eA_ ratio in epoch 1 (the current epoch) is given by *g*_Z_*/fg*_A_ (supplementary fig. S1, Supplementary Material online). The *N*_eZ_*/N*_eA_ ratio in epoch 2 was 0.50 (bootstrapping 95% CI: [0.34, 0.60]), significantly lower than the expected null value of 0.75, whereas that in epoch 1 was 0.72 (bootstrapping 95% CI: [0.46, 0.90]), not significantly different from 0.75. Likelihood ratio tests comparing the full model to a reduced model with a fixed *N*_eZ_*/N*_eA_ ratio of 0.75 also showed that the reduced model was significantly less likely than the full model (*P *=* *5.62 ×10^−41^). Because epoch 2 is the ancestral epoch, representing a much longer timescale than epoch 1, that *N*_eZ_*/N*_eA_ was estimated to be 0.50 suggests that *N*_eZ_ is likely to be lower than the null expectation for a substantial period of time during the evolution of the great tit. This may explain the evidence for relaxed purifying selection on the Z reported below (table 2).

**Table 1:** MLEs and Bootstrapping 95% CIs for Parameters of the 2-Epoch Model

Loci	θ	*f*	*g*	τ	ε
Z chromosome	0.00259	–	2.40	0.323	0.108
95% CI: lower/upper	0.00244/0.00268	N/A	2.24/2.58	0.269/0.437	0.0995/0.120
Autosomes	0.00204	1.99	1.68	0.323	0.112
95% CI: lower/upper	0.00139/0.00247	1.65/2.97	1.62/1.73	0.269/0.437	0.111/0.115

Note.—θ for the Z chromosome (Z) and the autosomes (A) is defined, respectively, as 4 *N*_eZ_*u*_z_ and 4 *N*_eZ_*u*_A_, where *N*_eZ_ is the effective population size of Z in the ancestral epoch and *u*_Z_ (*u*_A_) is the mutation rate per site per generation on Z (A). Note that θ is defined using *N*_eZ_ for both Z and A. *f* measures the ratio in the effective population size between A and Z in the ancestral epoch. The parameter *g* measures population size change, with *g *>* *1 signifying population expansion. Z and A are allowed to have different *g*. τ = *T*/(2*N*_eZ_), where *T* is the time (in generations) to the population size change event. ε is the polarization error parameter. A graphical representation of the model can be found in supplementary figure S1, Supplementary Material online.

**Table 2:** MLEs and Bootstrapping 95% CIs for the Parameter in the *anavar* Model with Two Site Classes

Loci	Neu θ	Sel_1_ θ	Sel_1_ γ	Sel_2_ θ	Sel_2_ γ	α	ω_ɑ_
Z chromosome	2.47×10^−3^	2.08×10^−3^	−195	3.90×10^−4^	−1.64	0.69	0.14
95% CI: lower/upper	2.14×10^−3^/2.95×10^−3^	1.80×10^−3^/2.51×10^−3^	−402/−115	2.66×10^−4^/4.95×10^−3^	−2.59/0.918	0.32/1.0	0.06/0.20
Autosome	3.65×10^−3^	3.12×10^−3^	−167	5.38×10^−4^	−2.58	0.85	0.183
95% CI: lower/upper	3.56×10^−3^/3.74×10^−3^	3.02×10^−3^/3.19×10^−3^	−176/−144	4.98×10^−4^/5.64×10^−4^	−2.76/−2.84	0.83/0.86	0.177/0.184

Note.—Ancestral repeats were used as the neutral reference. Z-linked and autosomal polymorphism data were analyzed separately to obtain estimates of the DFE for 0-fold variants. θ (4 *N*_e_*u*) is the per site scaled mutation rate, and is assumed to be constant across sites in a data set. γ (4 *N*_e_*s*) is the population scaled selection coefficient. Under a model with two site classes, these assumptions mean that *neu* θ = *sel*_1_ θ + *sel*_2_ θ, where *neu* θ is the scaled mutation rate per neutral site, *sel_i_* θ = *p_i_* × *neu* θ, and *p_i_* is the proportion of new 0-fold mutations with fitness effect *sel_i_* γ (*i *=* *1, 2). α is the proportion of 0-fold substitutions fixed by positive selection, and ω_ɑ_ is the (relative) rate of adaptive substitution at 0-fold sites. MLEs of the polarization error rate are shown in supplementary table S6, Supplementary Material online.

In agreement with the Tajima’s *D* statistic, there was also evidence of a population expansion (*g *>* *1), and that this has been more pronounced on the Z chromosome. The bootstrapping 95% CIs suggest that *g*_Z_ is significantly greater than *g*_A_, providing evidence for recent sex-biased demographic changes, which in turn leads to an increase in *N*_eZ_*/N*_eA_ in the current epoch.

Likelihood ratio tests comparing the full model to a reduced model with an equal mutation rate between loci showed that the reduced model was significantly less likely than the full model (*P *=* *0.0282). The bootstrapping 95% CIs for the mutation rate barely overlap between loci, further suggesting that the Z may have a higher mutation rate than the autosomes. Define β = *u*_m_/*u*_f_, where *u*_m_ and *u*_f_ are the mutation rate in the male and female germline, respectively. The Z chromosome spends one-third of the time in the female germline and two-thirds in the male one, whereas the autosomes spend an equal amount of time between the two. We can derive that *u*_Z_/*u*_A_ = (2 + 4β)/(3 + 3β). Equating this to the ratio reported in table 1, we obtain an estimate that β = 9.46. Bootstrapping suggests that β is significantly >1 (*P *<* *0.05). However, this estimate is highly variable, with the lower bound of the 95% CI being 1.37 (note that we were unable to obtain an upper bound because values in the upper tail of the distribution of *u*_Z_/*u*_A_ went above the maximum value of 4/3, corresponding to β = infinity). To gain further insight, we carried out a separate calculation by equating *u*_Z_/*u*_A_ = (2 + 4β)/(3 + 3β) to 0.078/0.068, the observed Z-to-autosome divergence ratio in ancestral repeats (fig. 1*a*). The result was β = 2.64 (95% CI = [2.13, 3.25]). Thus, these semi-independent data (i.e., polymorphism within great tits and substitutions along the great tit lineage) suggest that the mutation rate is higher in males than females.

### Estimating the Efficacy of Selection

Using SNPs in ancestral repeats as the neutral reference, we obtained MLEs of the DFE and scaled mutation rates from *anavar* (table 2). The majority of new mutations on both the Z chromosome and the autosomes were deleterious rather than beneficial, shown by negative selection coefficients for selected classes of site. Of these, the proportion that was strongly deleterious (*sel*_1_θ*/neu*θ) was 0.84 on the Z chromosome and 0.85 on the autosomes. The MLEs of the scaled selection coefficient (γ) for these sites were well below −100, meaning that they contribute little to polymorphism and divergence. The remaining proportion of mutations was nearly neutral. The Z chromosome has a significantly smaller γ for these sites than the autosomes (bootstrapping *P *<* *0.05). This is consistent with *N*_eZ_ < *N*_eA_ and suggests reduced efficacy of purifying selection on the Z chromosome.

To test whether there is evidence that the efficacy of positive selection is higher on the Z, as some theories have predicted (see Introduction), we estimated the proportion of 0-fold substitutions fixed by positive selection α, as well as the relative rate of adaptive substation ω_a_ (Gossmann et al. 2012). α was 0.69 (bootstrapping 95% CI: [0.32, 1.0]) on the Z chromosome and 0.85 (95% CI: [0.83, 0.86]) on the autosomes. ω_a_ was 0.14 (bootstrapping 95% CI: [0.08, 0.22]) on the Z chromosome and 0.183 (95% CI: [0.177, 0.184]) on the autosomes. These results suggest that there is no significant difference in the efficacy of positive selection between the Z chromosome and the autosomes, and if anything, positive selection may be more effective on the autosomes (as suggested by the nominally higher α on the autosomes).

We repeated the above analysis using 4-fold sites as the neutral reference (supplementary table S4, Supplementary Material online). In keeping with evidence for purifying selection acting on some of these sites reported earlier, the estimated strength of purifying selection in the DFE was lower (see Barton and Zeng 2018 for a discussion of this behavior). As a result, the estimate of α was lowered to 0.55 (bootstrapping 95% CI: [0.30, 0.96]) on the Z chromosome and 0.66 (bootstrapping 95% CI: [0.61, 0.71]) on the autosomes. Similarly, ω_a_ was 0.14 (bootstrapping 95% CI: [0.07, 0.22]) on the Z chromosome and 0.15 (bootstrapping 95% CI: [0.13, 0.16]) on the autosomes. On the other hand, when we assumed that the DFE followed a gamma distribution and used 4-fold sites as the neutral reference, the proportion of weakly deleterious 0-fold mutations with γ < −2 was 0.079 and 0.083 on the Z chromosome and the autosomes, respectively. This is qualitatively the same as the result presented earlier, although the difference is not significant. However, the gamma distribution may not be an adequate description of the underlying DFE and its use could compromise statistical power (Kousathanas and Keightley 2013). Based on the gamma DFEs, estimates of α for the Z chromosome and the autosomes were 74% and 71%, respectively, and were not significantly different. Overall, these results are in agreement with those based on ancestral repeats and shown in table 2 in that they also suggest there is no significant difference in the efficacy of positive selection between the Z chromosome and the autosomes.

## Discussion

### Mixed Support for Faster Divergence on the Z Chromosome

Several studies in birds have found significantly greater divergence on the Z chromosome than the autosomes in both protein coding and neutrally evolving sequences (Borge et al. 2005; Mank et al. 2007; Mank, Nam, et al. 2010; Mank, Vicoso, et al. 2010; Wang et al. 2014; Wright et al. 2015; Xu, Auer, et al. 2019; Xu, Wa Sin, et al. 2019). Here, we detected a significantly higher rate of divergence at the putatively neutral ancestral repeats. This is consistent with a higher mutation rate in the male germline. Our divergence-based estimate of the male-to-female mutation rate ratio (*u*_m_*/u*_f_) is 2.64, which is within the range previously observed in birds and a variety of other organisms (supplementary table S5, Supplementary Material online). Our model-based analysis of polymorphism data on ancestral repeats also points to a higher mutation rate on the Z chromosome (table 1). Although the scarcity of polymorphic sites (relative to fixed differences) means that the estimate is rather noisy, the polymorphism data are semi-independent of the divergence data. The fact that both approaches lend support to *u*_m_*/u*_f_ being >1 adds credence to the result. Put together, our estimates and those shown in supplementary table S5, Supplementary Material online, suggest that male-driven evolution may be rather ubiquitous.

Our evidence of a higher rate of divergence in coding regions on the Z chromosome is less conspicuous. Consistent with previous studies in birds (Borge et al. 2005; Mank et al. 2007; Mank, Nam, et al. 2010; Mank, Vicoso, et al. 2010; Wang et al. 2014; Wright et al. 2015; Xu, Auer, et al. 2019; Xu, Wa Sin, et al. 2019), *d*_0_*/d*_4_ is significantly higher on the Z chromosome in the great tit genome. However, this result seems to be mainly driven by a significantly lower *d*_4_ value on the Z, with the rate of divergence at 0-fold sites being very similar between the two types of chromosomes (fig. 1). When using ancestral repeats as the neutral reference, *d*_0_*/d*_AR_ is nominally lower on the Z chromosome (fig. 1*c*). Our observation that some of the 4-fold sites in the great tit genome may be subject to selective constraints is consistent with findings in an earlier study of several other avian genomes (Künstner et al. 2011). This makes *d*_0_*/d*_4_ hard to interpret and suggests that caution should be taken when using synonymous sites as the neutral reference. It is unknown what may be the causes of selective constraints at 4-fold sites. Evidence for selection on codon usage bias appears to be equivocal in birds (Rao et al. 2011; Galtier et al. 2018). It will be of interest to test whether other factors such as selection on exonic splice enhancers are involved (Chamary et al. 2006; Savisaar and Hurst 2018).

### Strong Evidence of a Low *N*_eZ_/*N*_eA_ Ratio

The Z chromosome to autosome effective population size ratio (*N*_eZ_*/N*_eA_) is known to be an important parameter in studies of faster-Z evolution (Vicoso and Charlesworth 2006; Charlesworth 2009; Ellegren 2009; Mank, Vicoso, et al. 2010). In this study, the Z-to-autosome diversity ratio is significantly lower than the null value of 0.75, regardless of whether 4-fold sites or ancestral repeats were analyzed. This is corroborated by our model-based analysis, which suggests the long-term *N*_eZ_*/N*_eA_ in the great tit is only 0.5, significantly <0.75 (table 1).

Studies on Z chromosomes in other species, and especially birds, have found similar patterns, with *N*_eZ_*/N*_eA_ ratios consistently being lower than expected (Ellegren 2009; Mank, Nam, et al. 2010; Mank, Vicoso, et al. 2010; Corl and Ellegren 2012; Oyler-McCance et al. 2015; Rousselle et al. 2016). In contrast, studies on the X chromosome have reported multiple instances where *N*_eX_*/N*_eA_ is larger than expected (Ellegren 2009; Mank, Vicoso, et al. 2010; Charlesworth et al. 2018). This discrepancy is usually attributed to the prevalence of polygyny in nature (Ellegren 2009; Webster and Wilson Sayres 2016). Because the Z chromosome spends 2/3 of its time in males, polygyny serves to lower *N*_eZ_*/N*_eA_. In support of this, in a recent study of multiple bird species, it was found that most polygynous species had lower *N*_eZ_*/N*_eA_ than monogamous species (Corl and Ellegren 2012). Great tits are one of the more monogamous passerine species, although some polygyny does occur (Björklund and Westman 1986; Dhondt 1987; Gohli et al. 2013). Our Z-to-autosome diversity ratio of 0.71 on ancestral repeats is close to the value of 0.69 observed in red-necked phalaropes (*Phalaropus lobatus*), a monogamous species (Corl and Ellegren 2012). However, there is evidence of population expansion in red-necked phalaropes (Corl and Ellegren 2012), and the authors did not use a model-based approach to infer possible changes in *N*_eZ_*/N*_eA_. Our analysis suggests that the population size expansion in great tits may have been driven by sex-biased demography, as it is accompanied by a shift in the *N*_eZ_*/N*_eA_ ratio. Thus, it is of interest to apply the model-based approach to a wider array of species with different mating systems to further clarify the relative contribution of mating system, sex-biased demography, and mutation rate variation to the Z-to-autosome diversity ratio.

### No Evidence of More Effective Selection on the Z Chromosome

Several theories predict that positive selection may be more effective on the Z chromosome due to the increased expression of recessive mutations (Charlesworth et al. 1987; Vicoso and Charlesworth 2006). However, the present study finds no evidence of this in the great tit. Both the proportion of mutations fixed by positive selection and the rate of adaptive substitution relative to the neutral rate did not differ significantly between the Z chromosome and the autosomes. If anything, selection may be more effective on the autosomes due to their larger effective population size. These results are consistent with other studies of Z chromosomes, particularly in birds, which also found no evidence that positive selection is more effective on the Z chromosome (Mank, Nam, et al. 2010; Wang et al. 2014; Wright et al. 2015; Rousselle et al. 2016; Xu, Wa Sin, et al. 2019), although there are examples of accelerated adaptive substitutions in *Lepidoptera* (Sackton et al. 2014; Pinharanda et al. 2019). Instead, the lower *N*_e_ has consistently led to increased drift and relaxed purifying selection on the Z chromosome (Mank, Nam, et al. 2010; Wright et al. 2015). In contrast, studies of X chromosomes have found evidence for more effective positive selection in several species (Carneiro et al. 2012; Hvilsom et al. 2012; Meisel and Connallon 2013; Kousathanas et al. 2014; Charlesworth et al. 2018). The reason for this discrepancy is unclear. For instance, the *N*_eX_*/N*_eA_ ratio is >0.75 in several *Drosophila* species where higher efficacy of positive selection has been reported (Meisel and Connallon 2013; Charlesworth et al. 2018). However, in the house mouse (*Mus musculus castaneus*), faster-X adaptive evolution was also observed, despite its π_X_/π_A_ ratio being 0.58, significantly <0.75 (Kousathanas et al. 2014). An interesting avenue for future investigation is to apply the *VarNe* model to these different systems. This will provide information about the *N*_eX_*/N*_eA_ or *N*_eZ_*/N*_eA_ ratio over different timescales (i.e., long- vs. short-term; table 1), which should in turn help us tease apart the contribution of *N*_e_ to the evolution of sex chromsomes.

### The Importance of Considering Other Complicating Factors

In addition to the aforementioned confounding effects of selection on synonymous sites and recent demographic changes, comparisons between the Z chromosome and autosomes can also be complicated by several other factors. First, although the Z chromosome may have a smaller effective population size than the autosomes, hemizygosity means that purifying selection against partially recessive deleterious mutations should be more effective on the Z (Charlesworth et al. 1987). This increase in the efficacy of selection may partially offset the reduction in the effective population size. Empirical evidence of this effect has been reported in two satyrine bufferflies, by comparing genes with male-biased, unbiased, and female-biased expression patterns (Rousselle et al. 2016). It will be of interest to carry out similar analyses in a larger array of species with good quality transcriptome data to test the generality of this observation.

The size of the great tit’s Z chromosome is 74.5 Mb, making it one of the macrochromosomes. Here, we have used data from all autosomes to increase the statistical power of our model-based analyses, on the basis that our autosomal data sets are dominated by data from macrochromosomes (chromosomes 1–12), such that summary statistics calculated on autosomal macrochromosomes alone are very similar those based on all the autosomes (supplementary table S3, Supplementary Material online). Nonetheless, macrochromosomes and microchromosomes in avian genomes are typically different in, for example, recombination rate, gene density, and GC content, which are known to modulate sequence evolution via processes such as linked selection and GC-biased gene conversion (Ellegren 2010; Bolivar et al. 2016; Corcoran et al. 2017). It will be interesting to carry out detailed research into how these factors contribute to the observed differences between the Z chromosomes and autosomes.

For instance, recombination rate, which varies significantly among the chromosomes in the great tit genome (van Oers et al. 2014), is an essential modulator of the strength of linked selection (Charlesworth 2012; Cutter and Payseur 2013). To understand to what extent the low long-term *N*_eZ_*/N*_eA_ reported here is due to linked selection, it will be necessary to obtain information on parameters that are currently poorly understood (e.g., the rate of recurrent sweeps and the DFE of new beneficial mutations). Although a detailed analysis is beyond the scope of this article, it is possible to obtain some suggestive information by carrying out calculations based on the following simplifying assumptions: 1) background selection is the predominant form of linked selection, 2) sites subject to deleterious mutation and selection are distributed uniformly across a chromosome, and 3) neutral diversity is calculated on variants far away from the edges of the chromosome. Under these assumptions, the effective population size is approximately *N*_e_ = *B* × *N*_e0_ = exp(−2*U*/*M*) × *N*_e0_, where *N*_e0_ is the effective population size in the absence of background selection, *U* is the “haploid” deleterious mutation rate for the chromosome, and *M* is the map length of the chromosome (Hudson and Kaplan 1995). In other words, *B* is a measure of the *N*_e_-reducing effect of background selection. *U* can be approximated by *u* × (*L*[coding] + *L*[conserved noncoding]), where *u* is the mutation rate per site per generation, *L*[coding] is the number of sites in coding regions, and *L*[conserved noncoding] is the number of sites in ultraconserved noncoding regions. We further assume that the autosomal mutation rate is *u*_A_ = 4.6 × 10^−9^, the same as the collared flycatcher (Smeds et al. 2016). We identified ultraconserved noncoding elements by using information on UCNEbase (Dimitrieva and Bucher 2013). Using the great tit linkage map (van Oers et al. 2014), we calculated *B* for the Z chromosome and autosomes. As can be seen in supplementary figure S2, Supplementary Material online, there is a clear negative correlation between *B* and chromosome size. Weighing the *B* values for individual autosomes by their sizes to mimic our use of data from all autosomes in the analysis, the autosomal average, denoted *B*_A_, is 0.94. For the Z chromosome, *B*_Z_ is 0.91, assuming that *u*_Z_ = 1.15 *u*_A_, as suggested by the difference in divergence rate in ancestral repeats (fig. 1*a*). Thus, *B*_Z_/*B*_A_ = 0.97. This suggests that background selection may only have a slightly larger *N*_e_-reducing effect on the Z chromosome, and hence may not be the sole reason for our observation that the long-term *N*_eZ_*/N*_eA_ is only ∼0.5.

It is known that linked selection can also distort the SFS (Cutter and Payseur 2013). This could in turn cause false inferences of recent changes in population size (Schrider et al. 2016), and could potentially contribute to our inference of a recent population expansion (table 1). The exploratory calculations shown above suggest that linked selection may have a relatively modest effect, and may affect both types of chromosomes to a similar degree (as measured by *B*_Z_ and *B*_A_). If this is true, then the significant difference between *g*_Z_ and *g*_A_ reported in table 1 is probably not entirely due to the SFS-distorting effect being much stronger on the Z chromosome. In addition, it is unlikely that the SFS-distorting effect would seriously affect our conclusion that the long-term *N*_eZ_*/N*_eA_ is significantly <0.75. As shown by Schrider et al. (2016), estimates of the ancestral *N*_e_ (i.e., that before recent demographic changes) using the SFS remain accurate, unless a large part of the genome is linked to a recent selective sweep where the selected mutation reached fixation immediately prior to sampling (e.g., >40%; see fig. 3 of Schrider et al. 2016), which does not seem very likely here. On the other hand, because the effects of background selection on the SFS tends to be weaker than sweeps (Zeng and Charlesworth 2011), its effect on the estimation of the long-term *N*_eZ_*/N*_eA_ is likely to be limited.

Our suggestion of the possibility of selective constraints on some 4-fold sites is based on the observation that, on both types of chromosomes, *d*_4_/*d*_AR_ < 1 (fig. 1*a*) and π_4_/π_AR_ < 1 (fig. 2*a*). A possible nonbiological explanation of these observations is that alignment quality is worse in ancestral repeats, leading to inflated divergence and polymorphism levels (Earl et al. 2014). Although this possibility cannot be ruled out completely, our main conclusions remain unchanged when 4-fold sites were used as the neutral reference (e.g., table 2 vs. supplementary table S4, Supplementary Material online). Similarly, in a recent analysis of the same data set wherein the DFE for insertion/deletion (INDEL) polymorphisms in coding regions of the great tit genome was inferred, the results were unchanged regardless of whether INDELs in ancestral repeat regions or noncoding regions were used as the neutral reference (Barton and Zeng 2019). In addition, as shown previously using the same data set, the diversity level for polymorphic INDELs in autosomal ancestral repeats π_indel_ = 0.00036, slightly lower than π_indel_ = 0.00038 in autosomal intergenic regions, whereas the nucleotide diversity level for these two types of genomic regions is π = 0.0043 and π = 0.0033, respectively (see table 1 in Barton and Zeng 2019). Thus, the difference in nucleotide diversity is probably not solely due to alignment issues caused by INDELs appearing more frequently in ancestral repeats. Finally, our observed *d*_4_/*d*_AR_ values are 0.77 and 0.96, for the Z chromosome and autosomes, respectively. They are within the range of values reported by Künstner et al. (2011) for the chicken (0.57), the turkey (0.70), and the zebra finch (0.76), or values reported by Eory et al. (2010) for hominids (0.73–0.78) and murids (0.88–0.89).

## Conclusion

Together, these results suggest that evolution of the Z chromosome in the great tit is characterized by a low effective population size, relaxed purifying selection, and a higher mutation rate in the male germline. There is no evidence of faster adaptive evolution. We also discovered that some 4-fold sites are probably under selective constraints, which, if left uncontrolled for, could potentially lead to biased results (e.g., those based on *d*_0_*/d*_4_). Furthermore, the *N*_eX_*/N*_eA_ or *N*_eZ_*/N*_eA_ ratio may be variable over time (e.g., table 1), and as a result, the π_X_/π_A_ or π_Z_/π_A_ ratio may not be the best measure of the ratio of *N*_e_ between sex chromosomes and autosomes. These results highlight the importance of considering multiple factors that can influence the rates of evolution of sex chromosomes and autosomes. 

## Supplementary Material

evaa044_Supplementary_Data
