# Supplementary material for: A Study of Faster-Z Evolution in the Great Tit (Parus major)
Source: Genome Biol Evol. 2020 Mar 2;12(3):210–22. doi: 10.1093/gbe/evaa044 (PMC7144363; doi:10.1093/gbe/evaa044)
Supplement: evaa044_Supplementary_Data [file evaa044_supplementary_data.pdf]

Supplementary Material

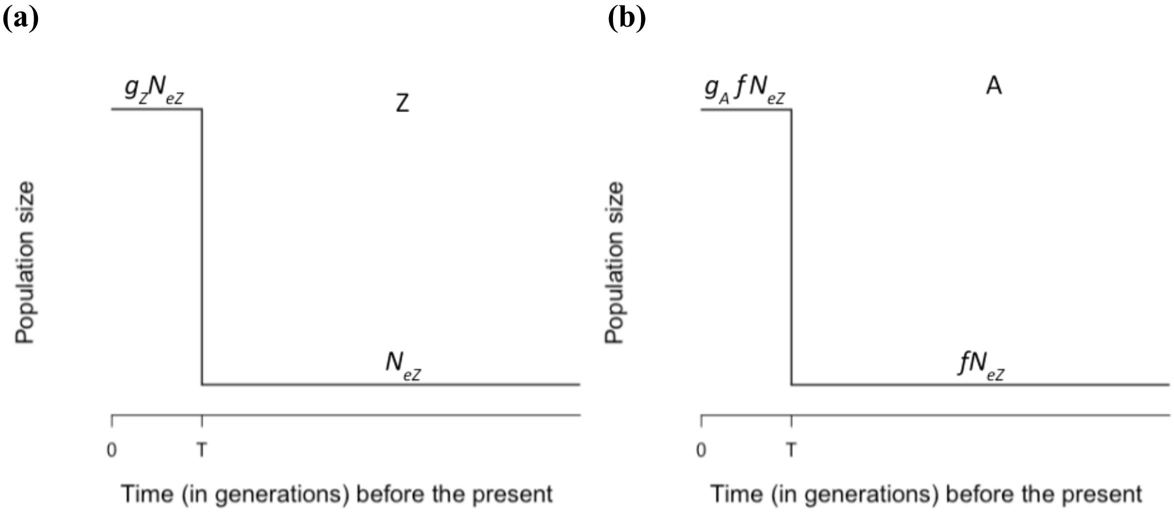

Figure S1 – Details of the 2-epoch model from *VarNe*. The ancestral population size of the Z and autosomes are N_{eZ} and $N_{eA} = fN_{eZ}$. Thus $N_{eZ}/N_{eA} = 1/f$. In the extant epoch, the population size of the two loci are $g_Z N_{eZ}$ and $g_A f N_{eZ}$, respectively. $\tau = T/2N_{eZ}$ is the scaled time to the population size change event. The scaled mutation rates are defined as $\theta_Z = 4N_{eZ}u_Z$ and $\theta_A = 4N_{eZ}u_A$, where u_Z and u_A are the mutation rate on the Z and autosomes, respectively. Because both θ_Z and θ_A are scaled by N_{eZ}, we have $u_Z/u_A = \theta_Z/\theta_A$.

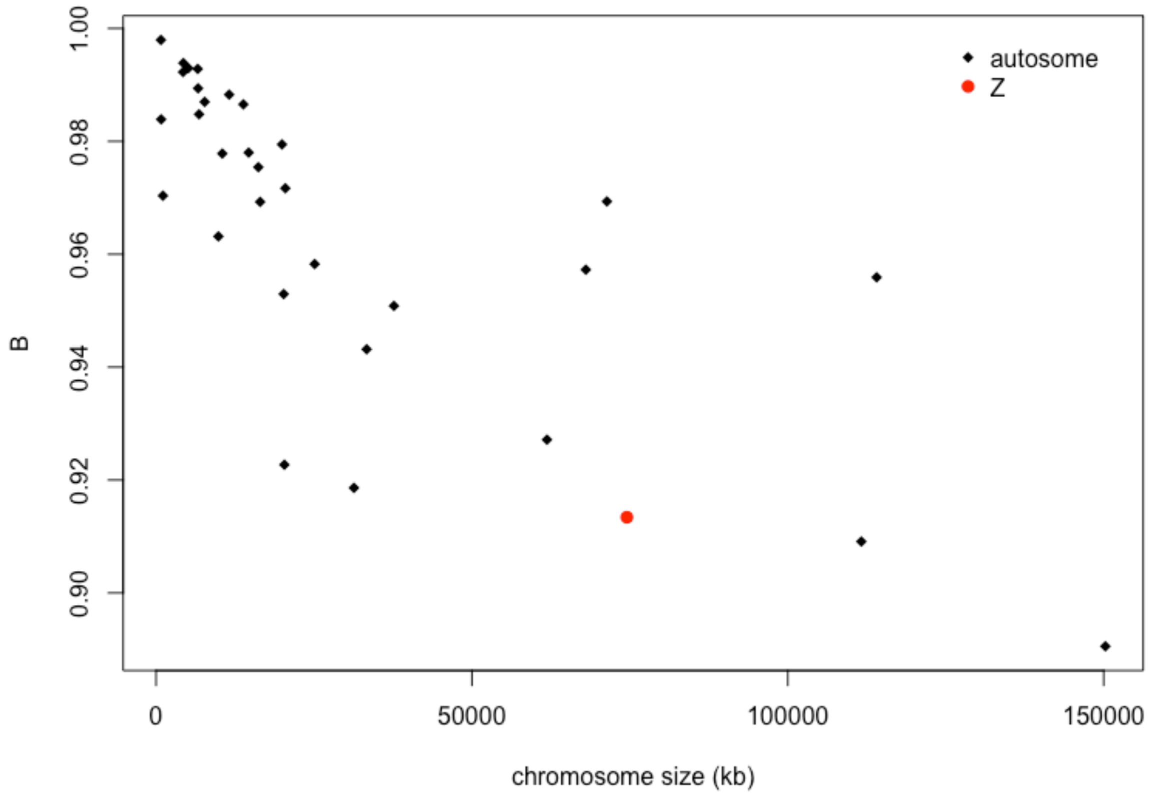

Figure S2. The predicted N_e-reducing effect of background selection on different chromosomes. *B* is defined in the main text.

Table S1. The numbers of sites analysed.

	Z			Autosomes		
	Length (Mb)	No. SNPs	Prop pol[2]	Length (Mb)	No. SNPs	Prop pol[2]
AR[1]	1.49	18,311	0.46	17.01	276,900	0.62
4-fold	0.189	1,735	0.33	3.54	53,360	0.40
0-fold	0.833	1,743	0.30	15.0	42,041	0.45

[1] Ancestral repeats

[2] Proportion of SNPs polarised

Note that a region was identified as an AR only if we had alignment coverage in all three species. As a result, we would not fail to polarise SNPs in ARs because we did not have alignment in any of the three species. In contrast, incomplete alignment coverage was the main reason why SNPs were not polarised in the other genomic regions. A similar observation was reported by Barton and Zeng (2019) in their analysis of INDEL polymorphisms in this dataset (see Figure S1 therein). These authors were able to polarise 38% of polymorphic INDELs within CDS regions on the autosomes, similar to the figures reported in this table.

Table S2. Assessing the performance of *anavar*'s polarisation error correction method.

We used parameter values realistic for the Z chromosome to generate simulated data. Specifically, the scaled mutation rate θ was set to 0.0025 per site, and the total number of sites sequenced was 475,625. The sample size was 20 alleles. The intensity of selection was measured by the scaled selection coefficient γ ($4N_e s$). The population size was assumed to be constant. Given θ and γ, unfolded SFSs were generated using the standard diffusion equations. For each polymorphic site, there was probability ε that its ancestral state was mis-inferred. When this happened, its frequency in the sample changes from i to $20 - i$ ($i \in \{1, 2, ..., 19\}$). The SFSs with polarisation errors added were then analysed by *anavar*. MLEs of the parameters shown in the table suggestion that the efforts of polarisation error can be adequately controlled for.

Case ID		θ	γ	ε
1	True	0.0025	0	0.05
	Mean MLE	0.00250	0.0	0.049
2	True	0.0025	0	0.1
	Mean MLE	0.00252	-0.0	0.10
3	True	0.0025	10	0.05
	Mean MLE	0.00251	9.9	0.056
4	True	0.0025	10	0.1
	Mean MLE	0.00250	10.2	0.097

Table S3. Comparison between summary statistics calculated on either all autosomes or macrochromosomes (chr 1 – 12).

Statistic	site type	Z chromosome	All autosomes	Macrochromosomes
π	AR	0.0030	0.0043	0.0043
	0 fold	0.00044	0.00058	0.00058
	4 fold	0.0021	0.0037	0.0035
θ_W	AR	0.0035	0.0046	0.0046
	0 fold	0.00059	0.00079	0.00078
	4 fold	0.0026	0.0042	0.0039
Tajima's D	AR	-0.52	-0.26	-0.27
	0 fold	-1.06	-1.10	-1.10
	4 fold	-0.72	-0.55	-0.50
Divergence	AR	0.078	0.068	0.070
	0 fold	0.016	0.015	0.014
	4 fold	0.060	0.065	0.063
d_0/d_4		0.26	0.22	0.22
d_0/d_{AR}		0.20	0.21	0.20
π_0/π_4		0.21	0.16	0.17
π_0/π_{AR}		0.14	0.14	0.13

Table S4. Parameter MLEs and 95% confidence intervals for the two site class model using 4-fold sites as the neutral reference. Where the population scaled mutation rate $\theta = 4N_eu$, the population scaled selection coefficient $\gamma = 4N_es$ (set to 0 for the neutral reference), the proportion of mutations fixed by positive selection a, and the rate of adaptive substitution ω_a. MLEs of the polarisation error rate are shown in supplementary

loci	*neu* θ		*sel₁* θ		*sel₁* γ		*sel₂* θ		*sel₂* γ		*a*		ω_a	
Z Chromosome	$2.14x10^{-3}$		$1.80x10^{-3}$		-137		$3.45x10^{-4}$		-0.629		0.55		0.14	
95% CI: lower / upper	*$3.52x10^{-4}$*	*$2.42x10^{-3}$*	*$3.50x10^{-4}$*	*$2.12x10^{-3}$*	*-254*	*-6.20*	*$2.17x10^{-6}$*	*$4.70x10^{-4}$*	*-1.84*	*10.9*	*0.30*	*0.96*	*0.07*	*0.22*
Autosomes	$2.9x10^{-3}$		$2.39x10^{-3}$		-125		$5.12x10^{-4}$		-1.51		0.66		0.15	
95% CI: lower / upper	*$2.82x10^{-3}$*	*$3.05x10^{-3}$*	*$2.31x10^{-3}$*	*$2.50x10^{-3}$*	*-142*	*-133*	*$4.82x10^{-4}$*	*$5.57x10^{-4}$*	*-1.85*	*-1.23*	*0.61*	*0.71*	*0.13*	*0.16*

Table S5. Estimates of u_m/u_f from different species and groups.

Group	Species	u_m/u_f	*Ref.*
Primates	-	4 - 6	Ellegren, 2007. Proc. R. Soc. [Biol]. 274:1-10.
Carnivores	-	3	Ellegren, 2007. Proc. R. Soc. [Biol]. 274:1-10.
Rodents	-	2	Ellegren, 2007. Proc. R. Soc. [Biol]. 274:1-10.
Birds	*F.albicollis, E.citrinella, L.svecica, P.trochilus, P.sibilatrix*	3.9 - 6.5	Ellegren and Fridolfsson, 1997. Nat. Genet. 17:182-184
	G.gallus, M.galopavo	2	Axelsson et al., 2004. Mol. Biol. Evol. 21: 1538-1547

Table S6.

Polarisation error MLE's and 95% confidence intervals for the two site class model, using either ancestral repeats (AR) or 4-fold sites as the neutral reference.

loci	*neutral ref.*	*neu ε*	*sel_1 ε*	*sel_2 ε*
Z Chromosome	AR	0.058	0.0	0.0
95% CI: lower / upper	-	*0.0 0.14*	*0.0 0.076*	*0.0 0.083*
Autosomes	AR	0.0	0.0	0.067
95% CI: lower / upper	-	*0.0 0.0*	*0.0 0.0*	*0.057 0.071*
Z Chromosome	4-fold	0.13	0.0	0.068
95% CI: lower / upper	-	*0.0 1.0*	*0.0 0.043*	*0.0 1.0*
Autosomes	4-fold	0.0	0.0026	0.033
95% CI: lower / upper	-	*0.0 0.0*	*0.0 0.0026*	*0.018 0.045*
